# Determining the Proportionality of Ischemic Stroke Risk Factors to Age

**DOI:** 10.3390/jcdd10020042

**Published:** 2023-01-23

**Authors:** Elizabeth Hunter, John D. Kelleher

**Affiliations:** ADAPT Centre, Technological University Dublin, Grangegorman, Dublin 7, D07 H6K8 Dublin, Ireland

**Keywords:** stroke, risk factors, non-proportional, risk prediction, logistic regression

## Abstract

While age is an important risk factor, there are some disadvantages to including it in a stroke risk model: age can dominate the risk score and lead to over- or under-predictions in some age groups. There is evidence to suggest that some of these disadvantages are due to the non-proportionality of other risk factors with age, e.g., risk factors contribute differently to stroke risk based on an individual’s age. In this paper, we present a framework to test if risk factors are proportional with age. We then apply the framework to a set of risk factors using Framingham heart study data from the NHLBI Biologic Specimen and Data Repository Information Coordinating Center to determine if we can find evidence of non-proportionality. Using our framework, we find that a number of risk factors (diastolic blood pressure, total cholesterol, BMI, sex, high blood pressure treatment) may be non-proportional to age. This suggests that testing for the proportionality of risk factors with age should be something that is considered in stroke risk prediction modelling and traditional modelling methods may need to be adjusted to capture this non-proportionality.

## 1. Introduction

Stroke is one of the leading causes of death and or disability in the world [1]. One of the methods to lower the population burden of stroke on society is through the use of stroke risk prediction models that are designed to identify individuals who might be at high risk of stroke. Extensive research has been conducted into such risk prediction models [2,3,4] and a large range of models have been created [5,6,7,8], including models that have been used in European guidelines for cardiovascular prevention [9].

In these risk prediction models, age is often considered as one of the main risk factors for stroke: the older a person is, the more likely they are to have a stroke [10]. Although the importance of the contribution of age to stroke risk is not in dispute, there are some problems that can arise from including age explicitly in a model. Because age is such a strong predictor for stroke, its contribution can dominate the risk score leading to under-prediction in younger age groups and over-prediction in older age groups [11]. The under- and over-predictions can have consequence for individuals, either causing unneeded stress in those who have a high risk simply because of their older age, or making an individual who has a low risk score due to age complacent despite having other risk factors for stroke that will lead to high risk as they age [12]. Hunter and Kelleher [13] find that when they create separate stroke risk prediction models for age groups instead of including age explicitly in the model they get better calibrated models and see differences in the feature importance of the risk factors by age group. One possible reason for this is that not all risk factors are proportional to age, meaning that as individuals age the ratio between age and the contribution of the risk factor is not constant. Although it could have a large impact on results, the impact of proportionality is not often considered in logistic regression models or other similar approaches that predict if stroke occurs within a time window.

These findings of a difference in importance and potential non-proportionality in risk factors by age are supported in the literature on stroke risk factors. There is evidence that across age groups all risk factors are not equivalent in predicting stroke risk: in particular, the contributions of BMI, HDL cholesterol levels, systolic blood pressure, blood glucose, and cigarette smoking to an individual’s stroke risk have been shown to decline with age [14]. A study looking at the influence of different stroke risk factors across age groups in Taiwan found that older stroke patients were more likely to have hypertension and diabetes while younger stroke patients were more likely to have obesity and high triglyceride levels [15]. Another study that looked at the association between blood pressure and stroke in a population in China found that the blood pressure values that lead to increased stroke risk vary by age [16]. Other research has looked at the impact of social factors on stroke risk and found that the effects of these factors change with age. A study on the social determinants of health, including race, education, income, zip code, and social isolation and their impact on stroke risk, found that, with each additional social determinant of health, the risk of stroke for an individual increased until the age of 75. After the age of 75, the social determinants of health did not appear to have an effect on an individual’s stroke risk [17]. Similarly, a study on socioeconomic factors and stroke found that lower socioeconomic status led to a higher risk of stroke in individuals under 75; however, in individuals over 75 a higher socioeconomic status led to a higher risk of stroke [18]. Looking primarily at younger ischemic stroke patients (40 and under), many of the risk factors are the same risk factors for ischemic stroke in older patients. However, the pathogenesis and the most common subtypes of ischemic strokes in younger patients often vary from that of older patients. Thus, the contribution of risk factors to stroke risk and any new unique risk factors in younger patients need to be considered [19]. Additionally, there are younger stroke patients who do not have any traditional stroke risk factors but instead might have conditions that are young-age-specific risk factors, for example, the use of combined oral contraceptive pills in younger woman. Research has also found that the early onset of some conditions, such as type 2 diabetes mellitus, might lead to a higher risk compared to later onset [20].

As there is significant evidence of the contribution of a risk factor changing with age, it needs to be determined if these changes are proportional or non-proportional. If a risk factor proportionally changes with age, the impact of this will likely be absorbed within the coefficient for age or can be mitigated using an interaction term with age. However, if the coefficients are not proportional with age, then an interaction term may not be enough to appropriately account for the change in risk factor contribution by age. Thus, it is necessary to have a method to determine if a risk factor is proportional to age before including the risk factor in a model and to determine if we actually see the non-proportionality of risk factors and age in practice. We propose a framework for determining if risk factors are proportional with age and use the framework to test a set of ischemic stroke risk factors to determine their proportionality to age.

## 2. Materials and Methods

In this section, we first discuss the details of the framework that we propose to use to assess the proportionality of risk factors. We then discuss the particular case study we use to test the framework and to test the hypothesis that not all risk factors are proportional by age.

### 2.1. Framework

To assess the non-proportionality of ischemic stroke risk factors by age, we propose a three-step framework. The steps are as follows:

1. Separate the data into a number of different age groups and create separate risk prediction models for each age group for each potential risk factor.
2. For each risk factor, check for differences in coefficients across the age-group-specific risk models for that risk factor.
3. If we see differences in coefficients, check for proportionality.

**Figure 1:**
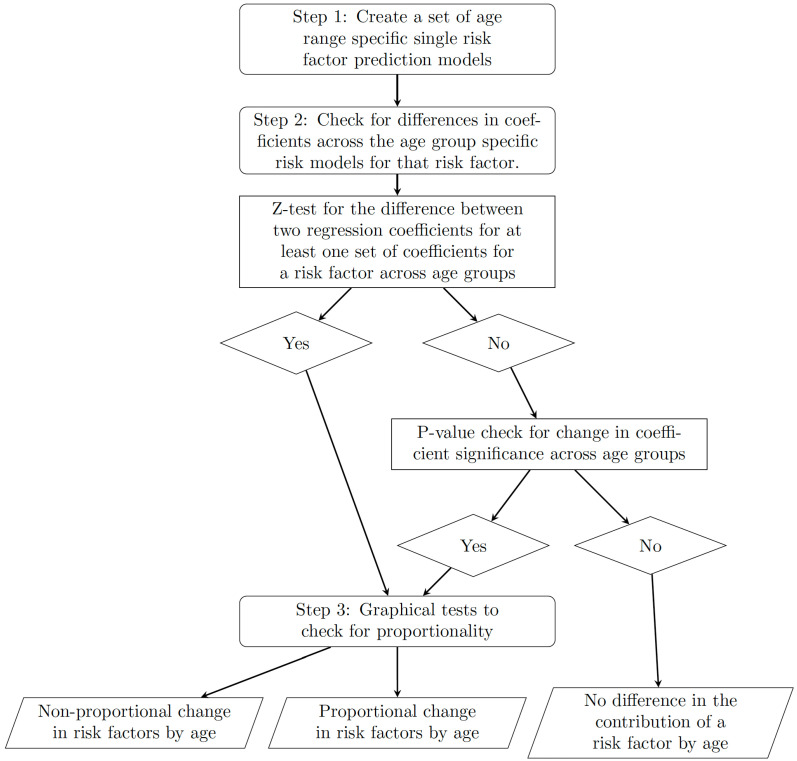
A diagram to show the process of the framework including the steps and decisions that can be made at each step.

The first step of the framework separates the data and creates risk prediction models, while the second and third steps are parts of the test for proportionality, with step two following on from step three. If, in the second step of the framework, no differences are found in the model coefficients for a given risk factor, then we do not move to step three for that risk factor as we have determined that the coefficient for the risk factor is likely to not change with age. The next sections provide a more detailed description of each step of the framework and Figure 1 is a process diagram of the different steps of the framework and the decisions that can follow from each step.

#### 2.1.1. Step 1: Separate Risk Prediction Models by Age Group

To look at the differences in the impact of risk factors by age group, we first separate our data set into predefined age groups. The age groups and the sizes of the age groups can be determined by the user and are based on domain knowledge. Once there are distinct data sets for each age, we fit a model for each risk factor and age group to predict the risk of stroke within a given time window. The type of model can be defined by the user but should be a risk prediction model that does not intrinsically include time in the model and does not consider when in the time window the stroke occurs as part of the individual’s risk. Logistic regression is an example of a model type that meets these criteria and so if we use logistic regression this will result in a separate single variable logistic regression model being created for each risk factor and age group.

#### 2.1.2. Step 2: Difference in Coefficients

If the contribution of a risk factor varies by age we should see differences in the observed coefficients for that risk factor (variable) across the different age models. Thus, the first step after the single variable logistic regression models are created is to determine if the coefficients for each age group differ. To check if the coefficients differ, we propose a two-step process. The first part of checking if the coefficients differ is to determine if the magnitudes of the coefficients are changing by age. This is conducted using a statistical test. We propose using a z-test for the difference between two regression coefficients. The formula for the statistical test from [21] based off of the work of [22] is the following:

(1) $$Z=\frac{b_{1}-b_{2}}{\sqrt{\left(SEb_{1}^{2}+SEb_{2}^{2}\right)}}$$

In the formula, $b_{1}$ and $b_{2}$ are the two coefficients being compared and $SEb_{1}$ and $SEb_{2}$ are the standard errors of the coefficients.

We propose that, in order to show there is a difference in coefficient magnitudes between age groups that might lead to a non-proportionality, we only need to find one difference in coefficient magnitudes. Thus, we should test the difference between the coefficients for the age groups that would appear to have the largest difference in magnitude. For example, if there are three age groups with coefficients 0.5, 0.51, and 0.7 it makes the most sense to test if 0.5 and 0.7 are statistically different as opposed to 0.5 and 0.51. However, if we do not find a difference in the first test that is run, we suggest performing a second statistical test. If the two coefficients with the second-largest difference in magnitudes have smaller standard errors than the two coefficients with the largest difference in magnitude, the coefficients should be tested. If the two coefficients with the second-largest difference in magnitudes have standard errors that are larger than the two coefficients with the largest difference in magnitudes then no more testing needs to be conducted and we can say that the coefficients’ magnitudes are not different. For example, if the standard errors for the coefficients 0.5, 0.51, and 0.7 are 0.1, 0.01, and 0.2, respectively, and we do not see a statistically significant difference when we test 0.7 and 0.5, we would test 0.51 and 0.7 because the sum of the standard errors (0.01 and 0.2) is less than the sum of the standard errors (0.1 and 0.2) for 0.7 and 0.5. If instead of 0.1, 0.01, and 0.2 the standard errors were 0.1, 0.3, and 0.2 we would not perform a second statistical test.

Performing the smallest number of statistical tests will help to mitigate the problem of multiple testing, where conducting more tests increases the chance of a type 1 error (false positive). As the framework is designed to assess a single risk factor, the problem of multiple testing could be resolved by reducing the number of age groups assessed. However, we do not want to restrict the number of age groups as this may have an impact on the results and could differ based on the data set. Additionally, while a smaller number of age groups might reduce the total number of tests, if the framework is being applied to a number of risk factors at the same time the problem of multiple testing might still apply. Another possible option to reduce the problems with multiple testing is through using a Bonferroni correction, where the significance level of a test is reduced by a factor based on the number of tests being performed. This in turn reduces the chance of a type 1 error. We have decided not to use a Bonferonni correction in this scenario for two main reasons. The first is that, while Bonferonni corrections reduce type 1 errors, they increase the chance of type 2 errors (false negatives) and, after consideration, we have determined that, within our framework in the second step, a smaller type 2 error is more preferable than a smaller type 1 error. This is because, if we falsely determine that there is a different in coefficient magnitudes, the third step of the framework is in place to determine if the changes in coefficients are proportional to age. If we had a false positive in step 2, step 3 is in place as a second test and will likely identify these cases as not proportional. However, if we had a high type 2 error and had a number of false negative tests, these risk factors would be considered to not change with age and would not be considered in step 3 for proportionality when in fact the risk factors do change with age. The second reason to not use a Bonferonni correction is that, depending on the number of age groups chosen and the number of risk factors being assessed, the number of tests performed could be large. For a single risk factor, the number of tests that need to be performed to test if each of the coefficients are different from every other coefficient is a combination of the number of age groups. For four age groups, six tests need to be performed, and for five age groups, 10 tests need to be performed. Thus, for the analysis in this paper, if all coefficients were tested for all seven risk factors, then there would be 42 statistical tests performed. While performing all of the tests might give a better overall idea of how the coefficients change with age, for the purpose of this framework we only need to determine that there is one difference in the coefficients. Thus, performing all 42 tests with a Bonferroni adjustment is not necessary.

If we observe a statistical difference in the magnitudes of the coefficients then for that risk factor we can move to step 3 of the framework and do not need the second check for difference in the coefficients. However, if we do not see a statistically significant difference in the magnitude of the coefficients we look at the *p*-value of the coefficients to determine if we see a change in coefficient significance across the age groups. A change in significance would suggest a change between age groups in the importance of the risk factor in predicting stroke. Thus, for the sets of models where we do not see a difference in coefficient magnitude across all the age groups, we determine if all of the age groups have statistically significant coefficients or if only some of the age groups have statistically significant coefficients.

If after step 2 we do not see a difference in either the magnitudes or the significance of the coefficients for a risk factor across age groups, then we assume that there is no difference in the contribution of the risk factor to an individual’s stroke risk. For that risk factor, we do not need to check proportionality by age in step 3.

#### 2.1.3. Step 3: Proportionality by Age

Differences in either the magnitudes or significance of the model coefficients for a single risk factor across age groups would suggest that there is a change in risk factor contribution between the age groups. However, it might not suggest a non-proportional change. It is possible that the coefficients will change by age group but will change proportionally. Thus, if we see a difference in either coefficient magnitudes and significance, we need to determine if this change is proportional. To test for proportionality by age we propose to use a graphical test. For the relationship between two variables to be proportional there should be a constant ratio between them; thus, a plot of the two variables should result in a linear relationship. To test for a linear relationship between age and the risk factors, we plot age on the x-axis and the magnitudes of the coefficients on the y-axis. For each coefficient, we consider the age to be the midpoint of the age group. For example, the x-coordinate for a coefficient for an age group that is 50–59 will be 54.5. If a line connecting the coefficients appears to be linear we will assume that the relationship is proportional. However, if there is a non-linearity we will assume that the relationship is non-proportional.

### 2.2. Case Study

For the case study we present in this paper, we predict the five-year risk of stroke for the age groups under 50, 50–59, 60–69, and 70 plus. We run seven different models considering one for each of four continuous variables: systolic blood pressure, diastolic blood pressure, total cholesterol, and body mass index (BMI) and three categorical variables: sex, atrial fibrillation, and treatment for high blood pressure. Although for our case study we use single variable logistic regression models predicting the risk of stroke in a five-year window, this method should work for any type of risk model that predicts stroke within a time window.

### 2.3. Data

For our analysis, we use data from the Framingham Heart Study, a longitudinal study designed to study the incidence and prevalence of cardiovascular disease and its risk factors. The study is one of the first long-term cohort studies of its kind and was designed to identify risk factors and characteristics that lead to cardiovascular disease. At the time of enrollment, participants have not yet developed symptoms of cardiovascular disease or had a heart attack or stroke. To create our modelling data sets we combine data from the Framingham-Cohort [23], Offspring [24], Third Generation, OMNI 2, and New Offspring [25] cohorts from the NHLBI. The study began in 1948 with the recruitment of 5208 men and women between the ages of 28 and 62 who lived in and around Framingham, Masssachusetts. After the initial recruitment, additional cohorts were included to increase the diversity of the patients in the study. The Framingham Offspring data include clinical exams from 5124 men and women between the ages of 5 and 70 who were the offspring or spouses of the offspring of the original Framingham cohort [24]. The Omni 1 cohort is made up of 507 men and women of African-American, Hispanic, Asian, Indian, Pacific Islander, and Native American origins, who were residents of Framingham or the surrounding towns when they were recruited in 1994 [24]. A total of 4095 men and women aged 19 plus with at least one parent in the Offspring cohort were recruited for the third generation cohort and spouses of those in the Offspring cohort not already recruited with at least one child in the third-generation cohort make up the New Offspring cohort [25]. To continue to increase the diversity of the data, the Omni 2 cohort enrolled 410 participants who were more ethnically diverse than the other cohorts [25]. Participants recruited into the Framingham Heart Study undergo clinical examinations and complete lifestyle and medical history questionnaires at regular intervals [23]. For more information on the data, patient characteristics, and data collection see https://biolincc.nhlbi.nih.gov/home/ (accessed on 25 January 2021).

We break down the longitudinal data into separate records per clinical exam as we are looking at short-term risk. Thus, the same person might appear in our modelling data sets more than once but each occurrence would be from a different clinical exam. We created a flag for clinical exams of individuals who suffered an ischemic stroke within five years of that clinical exam and remove a clinical exam from the data set where there is missing data or any of the continuous risk factors. Where there is missing data for our categorical variables, as most categorical variables are to identify a condition or treatment, we consider a missing variable equivalent to not having the condition. The resulting data set used for modelling contains 113,714 clinical exams.

To determine the non-proportionality of risk factors by age, we separate the data set into four data sets based on age: under 50, 50 to 59, 60 to 69, and 70 plus. As the data are unbalanced, with far more clinical exams where a stroke does not occur within five years, we select two controls for each stroke. The controls are matched on age and cardiovascular disease status and are selected from the set of clinical exams where the individual examined did not suffer a stroke within five years of the exam. The 1:2 (stroke:non-stroke) ratio is maintained across the four age groups.

## 3. Results

The following sections show the use of the framework to test for non-proportionality in risk factors for initial ischemic stroke using the case study described in the previous section. We first present the results for each age-specific model, then compare the magnitudes of the coefficients and the significance of the coefficients and finally test for proportionality by age.

### 3.1. Model Results

Table 1 shows the coefficients as well as the *p*-value and standard error for the coefficients for each of the four age models for the seven risk factors considered.

### 3.2. Coefficient Magnitude and Significance

After creating the models, the next step in the framework is looking at the magnitudes of the model coefficients and determining if they differ between age groups. Before running statistical tests, we conduct a spot check to see if the coefficients appear to change. From Table 1, we can see that the coefficient for systolic blood pressure decreases over time with the highest coefficient in the model for the under-50 age group and then the lowest in the over-70 age group. Similarly, we see the coefficients for diastolic blood pressure decrease by age after staying the same between the under-50 and 50-to-59 models. The coefficients in the models with total cholesterol as the risk factor again decrease with age from 0.36 in the under-50 model to 0.07. In the BMI model, the coefficients remain the same for the under-50 and 50-to-59 models and drop in magnitude and then remain the same between the 60-to-69 and over-70 models. The coefficients for the models with sex as the risk factor decrease from the youngest age group to the 50–59 and 60–69 age groups and then increase in the oldest age group. For atrial fibrillation, we observe a consistent increase in the size of the coefficient with age and for high blood pressure treatment we appear to see a decrease in the magnitude of the coefficient over time.

As our visual check of the magnitudes shows that there might be a difference between age groups we then conduct the statistical tests. Table 2 gives the statistical results for the magnitude checks for each of the risk factors considered. For each risk factor, the table shows the two age groups whose coefficients are compared in the test and in brackets the coefficient for that age group. (The coefficents compared are those with the largest difference in magnitude.) Then, the z-statistic is calculated using the formula in Section 2.1.2. We use the z-statistic to determine if the difference in magnitude is statistically significant, and if so what is the level of statistical significance (5% or 10%). If the z-statistic does not suggest significance and another set of coefficients is considered based on the criteria outlined in Section 2.1.2, then a second row for the same risk factor is included in the table.

From Table 2, we can see that three of the risk factors considered, systolic blood pressure, diastolic blood pressure, and atrial fibrillation, have at least one difference in the magnitudes of the coefficients across age groups at a 5% significance level. For these risk factors, the next part of step 2 does not need to be considered and we can move on to assessing their proportionality in step 3 of the framework. Three risk factors, total cholesterol, BMI, and sex, have a significant difference in coefficient magnitudes at the 10% level. As a significance level of 10% is not as strong as 5% for these risk factors, we will look at the changes in coefficient significance across age groups to further justify the change in the coefficients for these risk factors across age groups. Finally, there was one risk factor that did not have a significant difference in coefficient magnitudes across age groups. For high blood pressure treatment, we compared two sets of coefficients and did not find a significant difference in magnitudes and as there are no other combinations of model coefficients that would have smaller standard errors we conclude that there is no evidence for a significant difference in the magnitude of the coefficients across age groups for high blood pressure treatment.

As we saw some cases where the magnitudes of the coefficients were not statistically different across some of the age groups we also consider the changes in coefficient significance across age groups. Here, we only focus on the risk factors that did not have significant changes in coefficient magnitude from the previous step. The only risk factor that has no statistically significant differences between coefficients is high blood pressure treatment. For high blood pressure, we see changes in the significance of the individual coefficients through the *p*-values: the coefficient for the 60-to-69 model is the only coefficient that is significant at the 5% level. This suggests that even though the statistical tests in the previous section did not show evidence for changes in magnitude there appear to be changes in significance. Three other risk factors did not show significant differences in the magnitudes of the coefficients at the 5% level but did at the 10%: total cholesterol, BMI, and sex. Looking at the changes in the significance of the total cholesterol, we can see that for the under-50 age group the *p*-value is less than 0.05 so it is significant at the 5% level, the coefficients for the 50-to-59 and the 60-to-69 age groups are greater than 0.05 but less than 0.1, showing significance at the 10% level, and the coefficient for the 70-plus model is not significant. For BMI, the coefficients for the under-50 and 50-to-59 models are significant but the coefficients in the older two age models are not. The only age group that has a coefficient for sex that is significantly different than 0 at a 10% level is the over-70 age group. The changes in the coefficients in significance and magnitude across the age groups show that the contribution of each risk factor to an individual’s risk may not be proportional across ages. Thus, it is necessary to move on to step three of the framework and test for proportionality.

### 3.3. Proportionality

**Figure 2:**
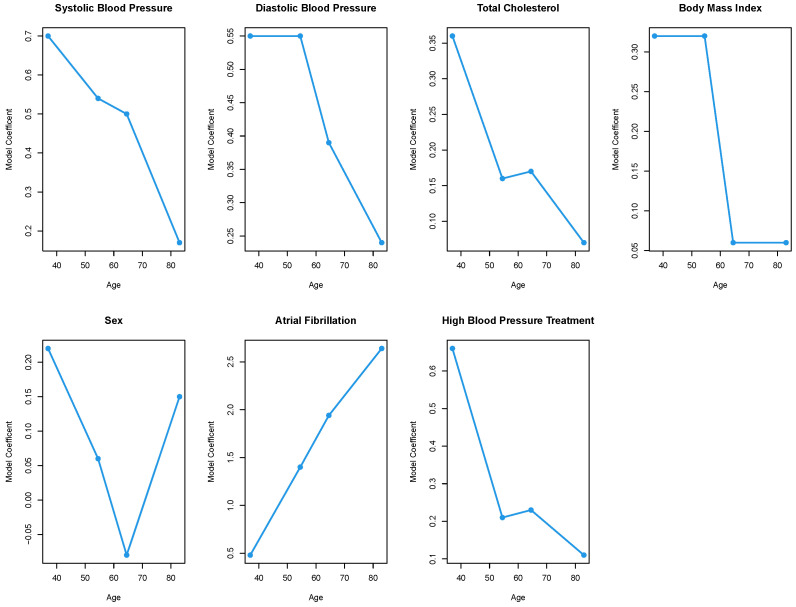
Plots of age versus model coefficients for each of the seven risk factors to test for risk factor proportionality by age. If the relationship between age and a risk factor is linear we consider the relationship to be proportional.

The final step of the framework is to plot the coefficients against age and determine if the relationship is linear. As all the risk factors investigated either have a change in magnitude, change in significance, or both, we look at all seven risk factors. Figure 2 shows the plots for each of the risk factors. If the relationship between age and the model coefficient for the risk factor is linear, then we consider the relationship proportional. Looking at the seven plots, we can see that systolic blood pressure and atrial fibrillation appear to have a relatively linear relationship suggesting that the contribution of the two risk factors changes proportionally with age. However, the plots for the other five risk factors (diastolic blood pressure, total cholesterol, BMI, sex, and high blood pressure treatment) do not appear linear. This would suggest that there is some non-proportional relationship between age and these risk factors.

## 4. Discussion

In this paper, we have presented a framework to assess the proportionality by age of risk factors for first time ischemic stroke. Our results show that not all risk factors appear to be proportional with age. We see that the coefficients for all risk factors change in magnitude, significance, or both and for five of our seven risk factors this change does not seem to be proportional with age. These results show the usefulness and importance of a framework to test proportionality.

There are two ways to consider the prediction of the event in a stroke risk prediction model: the model can predict if the event will occur within a time window, or, if the event occurs, when it will occur within the time window [26]. Predicting when an event occurs is considered time-to-event or survival analysis. In these methods, proportionality between risk factors is considered. A number of tests have been devised to determine if hazards are proportional over time [27,28,29] and, if the hazards are found to not be proportional over time, methods have been devised to take this into account in modelling, for example, creating stratified models [28]. However, there is little work considering how a risk factor for a regression or machine learning model that predicts if an event will occur within a time window might be impacted by proportionality. While the idea of proportional hazards in a survival analysis model considers the proportionality of the hazards of two risk factors over time, we propose that the equivalent in a logistic regression model, or any model that predicts if an event occurs within a time window, is to consider the proportionality between risk factors and age. Hunter and Kelleher [13] find that using a single model to predict stroke risk across all age groups results in over-predicting in the older age groups and under-predicting in the younger age groups. We believe those results are likely due to the non-proportionalities in risk factors that we have found in this paper.

While we present a framework to test for the proportionality of ischemic stroke risk factors to age, there are some limitations with our study. To compare the magnitudes of coefficients between age groups, if more than one test are needed per model this could lead to the problem of multiple testing and might result in finding significant differences where there are none due simply to chance. The problem with multiple testing will exist if only one variable is being tested but is compounded if multiple risk factors are tested as more tests will need to be conducted. This is why we suggest that only one statistically significant difference between age groups needs to be found to show that the magnitude of the coefficients might change with age. This, however, leads to a choice for the user and, if enough risk factors are tested, there is still the possibility that the results could be impacted by multiple testing. Although this is a limitation to the framework, the multiple-step framework for testing difference is designed to mitigate this risk. If we see a statistical difference through random chance, the proportionality still needs to be assessed through determining if the relationship between age and coefficient magnitude is linear.

Although we think that the method we have proposed in step three of the framework, plotting the magnitude of the coefficient by age, is suitable for determining proportionality, it is a visual method that might be impacted by individual bias. For example, in our analysis the plot of the coefficients for systolic blood pressure versus age appears nearly linear but the coefficient for the 60-to-69 age model slightly diverges from the line. As it is not far from linear, we assume a linear relationship, but others might not. Thus, future research may need to be conducted to develop more statistical and less visual tests for non-proportionality in regression coefficients. Additionally, we only use one data set and one set of age groups in the analysis. It is possible that we would see different results if we broke the data up into different age groups or if we considered a different population. The potential for differences is why we have presented a framework along with our case study. While different results might be found using different data, the same framework can still be applied to assess if risk factors are proportional to age.

Determining that non-proportionality is a problem in risk prediction then leads to the questions of how to deal with this problem. In addressing the non-proportionality of hazards in a survival analysis, stratified models are used that would break the population into a number of different groups. Hunter and Kelleher [13] use a similar method for logistic regression where they create stratified age models breaking the population into age groups and find that they get better predictions and better calibrated models compared to a non-stratified single model. However, these stratification methods have some drawbacks. By stratifying the data set, there are fewer cases to be considered for each model and if data are already sparse this could lead to problems such as overfitting. Additionally, if the models are stratified on a continuous variable such as age, this creates artificial cut points within the data that might lead to unlikely jumps in risk, for example, models stratified on age might predict a vastly different risk for a 49-year-old compared to a 50-year-old [30]. Thus, other methods beyond stratification should be considered in modelling stroke risk prediction if non-proportionality is detected.

Beyond modelling, determining that there is non-proportionality between age and other risk factors leads to another question regarding what are the clinical consequences of identifying and modelling non-proportionality between age and risk factors in a risk prediction model? Using a risk prediction model that takes this non-proportionality into account might lead to the better targeting of patients who are actually at high risk. For example, an 80-year-old patient who has a high risk in a model that does not take the proportionality of risk factors by age into account might have a lower risk in a model that does take the proportionality into account. This patient might be saved the stress of thinking they are high risk and potentially unnecessary treatments if risk factor proportionality to age is considered. Conversely, a 40-year-old who is low risk in a model that does not take the proportionality of risk factors by age into account might have a higher risk in a model that does. Not treating this younger patient due to an incorrect low risk prediction could lead to the patient having a stroke. Additionally, knowing that there are certain risk factors that contribute more to the short-term risk of a given age group could alter how much emphasis clinicians put on the risk factor for a given age group. For example, the coefficient for total cholesterol in the single variable logistic regression models is low in the over-70 age group. This suggests that total cholesterol might not be as important of a contributor to stroke risk in this group compared to other risk factors. Thus, if a patient over 70 presents to a clinician with high total cholesterol and high blood pressure, while reducing both risk factors would be ideal, if the clinician needs to focus on one they could focus on high blood pressure as the relationship between high blood pressure and stroke risk appears to be more important in the over-70 group.

## 5. Conclusions

Our results show that through using the proposed framework for identifying if risk factors in a logistic regression model are proportional to age we find that a number of primary ischemic stroke risk factors are not proportional. This non-proportionality would suggest that traditional risk models such as logistic regression that use age as a risk factor might need to be adjusted to take this non-proportionality into account. Although we apply our framework to ischemic stroke risk, this non-proportionality is likely to exist in other diseases where age is a dominant risk factor; thus, the framework has potential applications beyond stroke.

## Figures and Tables

**Table 1:** Coefficients, *p*-values, and standard errors for the single variable logistic regression models for each age group.

	Under 50	50 to 59	60 to 69	Over 70
**Systolic Blood Pressure**				
Coefficient	0.70	0.54	0.50	0.17
*p*-value	4.52 × 10^−6^	4.00 × 10^−11^	<2.00 × 10^−16^	2.27 × 10^−6^
Standard error	0.153	0.081	0.053	0.037
**Diastolic Blood Pressure**				
Coefficient	0.55	0.55	0.39	0.24
*p*-value	0.0001	5.11 × 10^−11^	9.32 × 10^−14^	8.6 × 10^−11^
Standard error	0.143	0.083	0.052	0.038
**Total Cholesterol**				
Coefficient	0.36	0.16	0.17	0.07
*p*-value	0.01	0.08	0.006	0.19
Standard error	0.143	0.090	0.063	0.050
**BMI**				
Coefficient	0.32	0.32	0.06	0.06
*p*-value	0.02	4.03 × 10^−5^	0.27	0.12
Standard error	0.136	0.078	0.050	0.037
**Sex (Male as Reference Category)**				
Coefficient	0.22	0.06	-0.08	0.15
*p*-value	0.42	0.72	0.41	0.05
Standard error	0.271	0.155	0.100	0.074
**Atrial Fibrillation**				
Coefficient	0.48	1.40	1.94	2.64
*p*-value	0.36	0.0002	9.59 × 10^−16^	<2 × 10^−16^
Standard error	0.523	0.381	0.241	0.155
**High Blood Pressure Treatment**				
Coefficient	0.66	0.21	0.23	0.11
*p*-value	0.15	0.31	0.03	0.15
Standard error	0.460	0.201	0.104	0.074

**Table 2:** Z-statistic and significance for the difference in two coefficients for each age group.

Risk Factor	Age Group 1	Age Group 2	Z-Statistics	Significant
Systolic Blood Pressure	under 50 (0.70)	70 plus (0.17)	3.37	Yes (5%)
Diastolic Blood Pressure	under 50 (0.55)	70 plus (0.24)	2.07	Yes (5%)
Total Cholesterol	under 50 (0.36)	70 plus (0.07)	1.958	Yes (10%)
BMI	under 50 (0.32)	70 plus (0.06)	1.88	Yes (10%)
Sex (Male as Reference Category)	under 50 (0.22)	60 to 69 (–0.08)	1.05	No
	60 to 69 (–0.08)	over 70 (0.15)	1.81	Yes (10%)
Atrial Fibrillation	under 50 (0.48)	70 plus (2.64)	3.97	Yes (5%)
High Blood Pressure Treatment	under 50 (0.66)	over 70 (0.11)	1.19	No
	60 to 69 (0.23)	over 70 (0.11)	0.99	No

## Data Availability

The data analysed in this study were obtained from the National Institute of Health (NIH) Biologic Specimen and Data Repository Information Coordinating Center (BioLINCC) website, and the following licenses/restrictions apply: these data sets must be requested from the NIH BioLINCC website. Requests to access these data sets should be directed to https://biolincc.nhlbi.nih.gov/studies/gen3/ (accessed on 25 January 2021).
